# The Iodine–Dextrin–Lithium Complex: Morphology, Antibacterial Activity, and Cytotoxicity

**DOI:** 10.3390/molecules30244822

**Published:** 2025-12-18

**Authors:** Daniil Shepilov, Seitzhan Turganbay, Ardak Jumagaziyeva, Tamara Bukeyeva, Dana Askarova, Didar Bolatova, Yerlan Doszhanov, Arman Zhumazhanov, Almagul Kerimkulova, Karina Saurykova, Aitugan Sabitov

**Affiliations:** 1 JSC Scientific Center for Anti-Infectious Drugs, Almaty 050060, Kazakhstan; shepilov2002@gmail.com (D.S.); turganbay.s@gmail.com (S.T.); bukeyeva_t@mail.ru (T.B.); askarova.dana08@gmail.com (D.A.); 2 Departement of Science, International IT University, Almaty 050040, Kazakhstan; dikobolatova.db@gmail.com; 3 UNESCO Chair in Sustainable Development, Al-Farabi Kazakh National University, Al-Farabi Ave., 71, Almaty 050040, Kazakhstan; doszhanov_yerlan@mail.ru (Y.D.); jumajanarman@gmail.com (A.Z.); 4 Department of Material Sciences, Nanotechnology and Engineering Physics, Satbayev University, Satbayev Str. 22a, Almaty 050013, Kazakhstan; almusha_84@mail.ru; 5 Institute of Combustion Problems, Almaty 050012, Kazakhstan; saurykova.karina@mail.ru; 6 Faculty of Natural Sciences, Kazakh National Women’s Teacher Training University, Almaty 050000, Kazakhstan

**Keywords:** dextrin, iodine, polymer–iodine complex, antimicrobial activity, cytotoxicity

## Abstract

A new iodine–dextrin–lithium complex (IDLC) was synthesized and structurally characterized as a hybrid supramolecular system combining antiseptic, stabilizing, and biocompatible components. The compound integrates iodine as the primary antimicrobial agent, lithium as a coordination and stabilization element, and dextrin as a biodegradable polysaccharide matrix enabling sustained release. Physicochemical analyses confirmed the formation of a uniform, thermally stable complex. Biological evaluation revealed strong bactericidal activity, with minimum bactericidal concentrations (MBCs) ranging from 1.95 to 15.63 µg mL^−1^ against both Gram-positive and Gram-negative pathogens, including multidrug-resistant *Staphylococcus aureus* and *Acinetobacter baumannii*. Cytotoxicity studies revealed moderate, concentration-dependent effects on human peripheral blood mononuclear cells (CC_50_ = 0.23–0.48 mg/mL; 11.7–24.4 μg I/mL) and low toxicity toward MDCK cells (CC_50_ = 10–20 mg/mL; 507–1014 μg I/mL), confirming a favorable safety profile. IDLC exhibited cytotoxic effects on tumor cell lines (HepG2, HeLa, AGS, K562, and H9) as well as on the normal MeT-5A cell line; however, the CC_50_ values are similar, and selectivity indices are close to 1, indicating no selective cytotoxicity toward tumor cells. Thus, IDLC demonstrates non-specific cytotoxicity at high concentrations, consistent with its iodine content. The research confirms that iodine can be effectively stabilized within a dextrin-lithium framework to yield a biologically active, thermally resistant complex, suitable for pharmaceutical use.

## 1. Introduction

The objective of modern pharmaceutical manufacturing is the development of not necessarily structurally intricate molecules, but more functionally flexible molecules—complex formulations comprising various bioactive constituents that exert targeted therapeutic effects through synergistic interactions. In the formulation of complex drug products, it is essential to consider both the individual pharmacological properties of each component and their conjoint synergistic potential [1].

Iodine exerts its antimicrobial activity predominantly via oxidative mechanisms, targeting vital cellular structures such as membrane lipids and proteins, resulting in damage to microbial membranes, protein denaturation, and metabolic inhibition [2]. The oxidative disruption of cell wall integrity by iodine leads to rapid microbial lysis and renders pathogens unable to mount effective resistance mechanisms. Iodine demonstrates efficacy against a diverse array of pathogenic microorganisms, including multidrug-resistant strains [3,4].

Lithium is frequently used as a stabilizing agent in pharmaceutical preparations due to its ability to reduce the degradation rate of active substances and extend their duration of action. In addition, lithium exhibits the ability to modulate cellular processes and acts as a membrane stabilizer, impairing the normal functioning of bacterial cells [5,6,7,8]. Its ability to interfere with microbial transport systems and enzymatic pathways positions lithium as a potentiator of antimicrobial action within combinatory systems.

Dextrin, a polysaccharide derivative utilized as a pharmaceutical adjuvant, serves multiple functions within the formulation. It enhances the solubility and bioavailability of APIs and acts as a colloidal stabilizer in aqueous formulations. Dextrin effectively encapsulates labile components such as iodine by preventing premature breakdown in biological systems, thus maintaining the chemical stability and pharmacological availability of the formulation [9,10,11,12].

The rational selection of iodine, lithium, and dextrin as components of the multicomponent therapeutic system is predicated not only on their individual bioactivities but also on their capacity for synergistic integration. Iodine serves as the principal antiseptic, while lithium enhances its activity and stabilizes the complex, and dextrin improves release and mitigates irritant effects.

Equally critical to the development of such a complex is the characterization of its physicochemical properties. These parameters determine the viability of the complex as a stable, efficacious therapeutic agent suitable for translational application across multiple delivery platforms.

## 2. Results and Discussion

### 2.1. Results of Quantum-Chemical Calculations

Studying the structure of a compound using the quantum-chemical method before the start of synthesis allows not only to predict chemical reactions but also to optimize the choice of synthesis conditions, such as temperature, catalysts, solvents, and other factors. Special attention is paid to the active centers of the molecule—atoms or their groups, which are key participants in chemical transformations.

**Figure 1:**
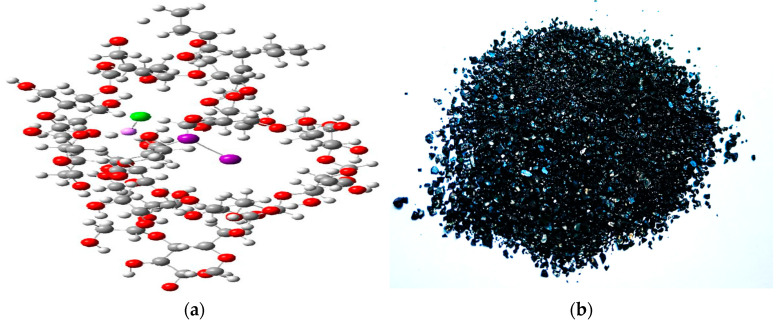
Molecular structure of the new compound IDLC, incorporating two dextrin rings (**a**), and the appearance of the obtained samples (**b**). Atoms are represented with the following color scheme: gray for carbon, red for oxygen, violet for iodine, green for lithium, and small white spheres for hydrogen.

The active centers of the iodine-containing complexes were modeled using quantum-chemical calculations employing density functional theory (DFT). The molecular structure of the new compound that was coded as IDLC, incorporating two dextrin rings (a), and the appearance of the obtained samples (b) are shown in Figure 1.

In the molecular structure images, atoms are represented with the following color scheme: gray for carbon, red for oxygen, violet for iodine, green for lithium, and small white spheres for hydrogen.

In complex III, the Li^+^ ion engages in non-covalent interactions with iodine and chlorine atoms, characterized by interatomic distances of 3.02 Å (Li-I) and 2.25 Å (Li-Cl), respectively. The lithium cation is additionally coordinated by an oxygen atom from the dextrin helix (Li-O = 2.22 Å), suggesting a chelating interaction. Molecular iodine within the complex demonstrates ambident behavior, functioning as an electron acceptor toward the chloride anion (I-Cl = 2.22 Å) and as an electron donor toward the lithium cation, as evidenced by the I-I bond length of 2.82 Å. The calculated complexation energy is ΔE = −843.27 kcal/mol.

The calculation results show that molecular iodine and triiodide can be embedded in two dextrin rings, which helps to stabilize their structure and improve their interactions with biological objects [13]. In particular, the interaction of iodine atoms with carbon and oxygen atoms of the dextrin ring through Van der Waals forces contributes to the structural stabilization of the assembly, whereas the absolute value of ΔE should not be considered a direct indicator of thermodynamic stability.

### 2.2. Physicochemical Properties of the Iodine-Containing Complex (IDLC)

The IDLC samples represent aqueous systems of coordination iodine complexes formed with multidentate carbohydrate ligands. In these systems, the active component is an iodine–polymer association that functions as a carrier matrix, providing a gradual and sustained release of molecular iodine. The physical characteristics of the obtained complexes—including color, yield, and melting or decomposition temperature—are summarized in Table 1. The synthesized IDLC exhibited a dark-grey appearance (Figure 1b). The water solubility of the complexes was evaluated using the flask method (for concentrations above 0.01 g/L) in accordance with the OECD Guidelines for the Testing of Chemicals (Water Solubility). Capillary electrophoresis (CE) analysis showed that the average iodide ion concentration in IDLC is 43.25 mg/L, while the lithium ion content is 6.78 mg/L (Table 2).

### 2.3. UV-Vis Spectral Analysis

**Figure 2:**
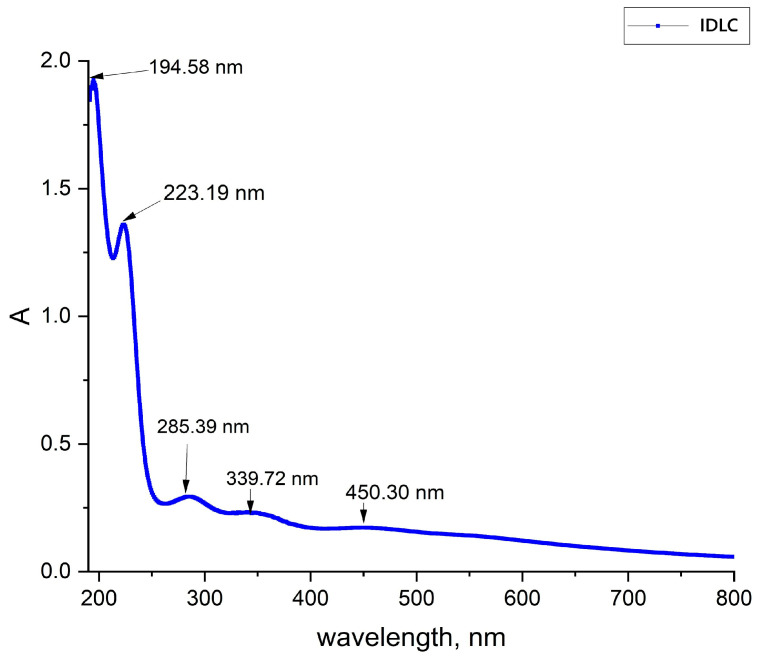
UV-vis spectrum of IDLC (0.025% solution).

Figure 2 presents the spectral characteristics of IDLC. The peaks at 194.58 nm and 223.19 nm correspond to the σ → σ∗ transitions, which are characteristic of ionic bonds such as Li^+^I^−^ and Li^+^Cl^−^ [14,15]. The lithium–chlorine bonds (Li^+^Cl^−^) contribute to the spectrum, causing additional absorption. The peak at 285.39 nm is associated with an n → π∗ transition occurring in iodine (I_2_) molecules. The peak at 339.72 nm is attributed to π → π∗ transitions taking place in the iodine molecular system or iodide complexes. The long-wavelength peak at 450.30 nm corresponds to molecular resonance of iodine or to the formation of its complexes.

### 2.4. TG Analysis

**Figure 3:**
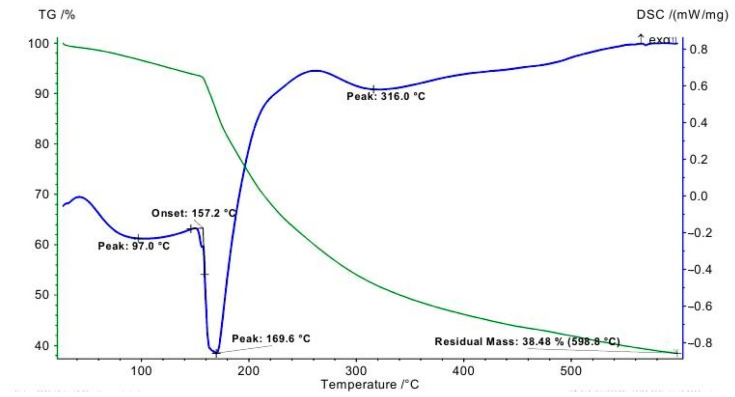
DSC (blue line) and TGA (green line) curves of IDLC.

The thermal behaviour of IDLC, which contains dextrin as a matrix component, was evaluated using thermogravimetric analysis (TGA) and differential scanning calorimetry (DSC), as presented in Figure 3. The melting point of the complex, observed at 169.6 °C on the DSC curve, is in good agreement with the value determined using a Gallenkamp variable heating device, thereby confirming the consistency of the data.

Thermal decomposition of the IDLC complex occurs in two main stages. Stage I takes place within the temperature range of 28–150 °C, corresponding to the release of physically adsorbed and structurally bound water molecules. This is accompanied by a mass loss of 8.13%, and the DSC curve displays a minor endothermic peak near 97.0 °C, which is consistent with the dehydration process. Stage II begins above 169.6 °C and is associated with the thermal degradation of the organic and iodinated components of the complex. A significant mass loss of 53.39% is recorded during this stage, indicating the breakdown of the dextrin-based polymeric framework and iodine-containing moieties. The DSC thermogram exhibits two prominent thermal events: an endothermic peak at 169.6 °C, attributed to melting, and a second event at 316.0 °C, which likely corresponds to further decomposition or structural rearrangement. After heating to 500 °C, the residual ash content was found to be 38.48%, suggesting nearly complete decomposition of the compound.

According to literature, native dextrin typically shows thermal degradation in the range of 250–350 °C, with no distinct melting point due to its amorphous and polymeric nature [16]. In the IDLC complex, the appearance of a sharp melting peak at 169.6 °C indicates a change in the thermal behavior of dextrin caused by specific interactions with lithium polyiodide species and structural reorganization within the complex. This shift suggests the formation of a more ordered supramolecular structure compared to free dextrin.

Overall, TGA/DSC analysis of IDLC demonstrates a well-defined thermal degradation profile and nearly complete mass loss, indicating high chemical purity and thermal stability of the compound under the experimental conditions.

### 2.5. ^1^H NMR Characterization

The ^1^H and ^13^C NMR spectra of the IDLC sample confirm the preservation of the polysaccharide backbone and reveal specific interactions between dextrin and iodine species, likely mediated by lithium ions. In the ^1^H NMR spectrum (Figure S1), a distinct signal at 5.239 ppm corresponds to the anomeric proton (H1) of α-1,4-glycosidic linkages, characteristic of glucosidic bonds in starch-derived oligosaccharides. Multiplets in the range of 3.1–3.7 ppm (at 3.108, 3.259, 3.479, 3.588, and 3.612 ppm) are assigned to protons at positions C2-C6 of the glucose units, while a signal at 3.689 ppm corresponds to methylene (CH_2_) groups at branching points, typically associated with α-1,6-linkages [3]. The broadness of these signals reflects polymer heterogeneity and possible supramolecular interactions. The ^13^C NMR spectrum (Figure S2) displays resonances for anomeric carbon atoms (C1) at 95.746 ppm (α-form) and 99.580 ppm (β-form), ring carbons (C2–C5) in the range of 68–78 ppm (e.g., 69.277, 71.137, 71.528, 72.673, 73.331, and 76.679 ppm), and the C6 carbon at 60.425 ppm, corresponding to the primary hydroxymethyl group [17]. The observed chemical shift deviations and signal broadening, compared to native dextrin, are attributed to weak ion–dipole or coordination interactions between polyiodide species and hydroxyl groups of the dextrin chain, potentially stabilized by lithium cations [18]. These spectral features support the formation of a structured iodine–polymer complex, as illustrated in Figure S3 (Supplementary Materials), and are consistent with earlier studies on similar iodine–polysaccharide systems [19].

### 2.6. XRD Analysis

**Figure 4:**
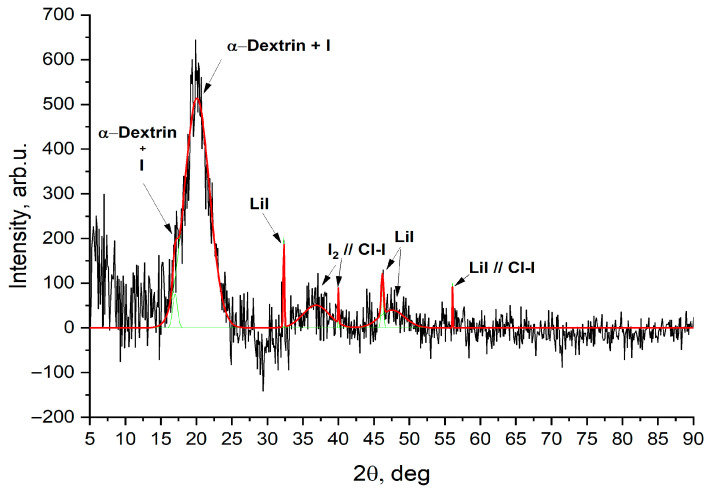
XRD pattern of the IDLC.

The XRD pattern of the synthesized sample reveals a combination of crystalline and amorphous features, reflecting the hybrid nature of the iodine–polysaccharide complex (Figure 4).

A broad peak centered around 2θ ≈ 20° corresponds to the amorphous halo of the polysaccharide backbone, which is typical for non-crystalline carbohydrate matrices such as dextrin. The slight shift and increased intensity of this peak compared to pure dextrin suggest partial structural ordering or intermolecular interaction due to iodine incorporation. Distinct crystalline reflections are observed at 2θ = 30.5°, 37.5°, 44.5°, 50.5°, and 55.5°, which match the reference diffraction peaks for lithium iodide (LiI) and confirm its crystalline presence in the sample. These peaks are sharp and well-defined, indicating high crystallinity of the LiI phase. Additional signals at 2θ ≈ 35–37° and 50–56° can be attributed to LiI-I_2_ and LiI-Cl-I associate phases, suggesting the formation of mixed halide–iodine interactions within the structure. The signal labeled as I_2_//Cl-I corresponds to a possible co-crystallization or complexation between molecular iodine and chloride–iodide species, stabilized by the Li^+^ cation.

The presence of combined reflections from LiI, elemental iodine, and polysaccharide-associated iodine phases indicates the formation of a semiorganic hybrid material, where iodine is partially complexed with the carbohydrate matrix and partially present in crystalline inorganic domains. This supports the proposed structure of a multicomponent iodine coordination complex, where iodine species exist both in bound and free (crystalline) forms.

### 2.7. SEM-Analysis Results

**Figure 5:**
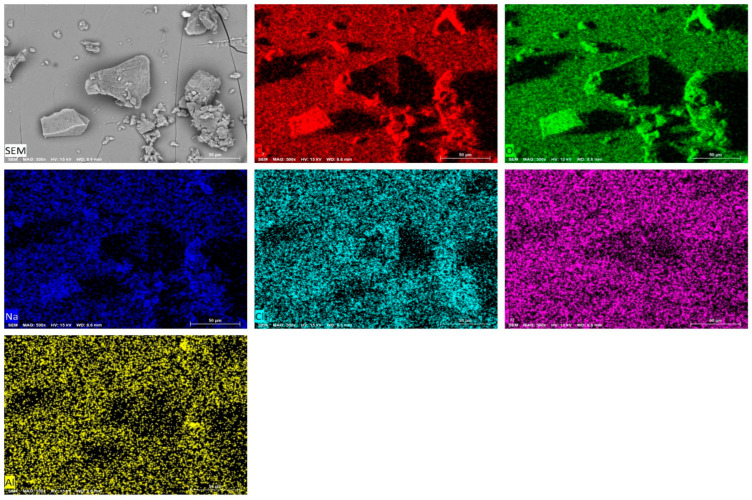
SEM images of the IDLC. C, D, Na, Cl, I and Al is the modes to detect of elements in sample by EDX.

**Figure 6:**
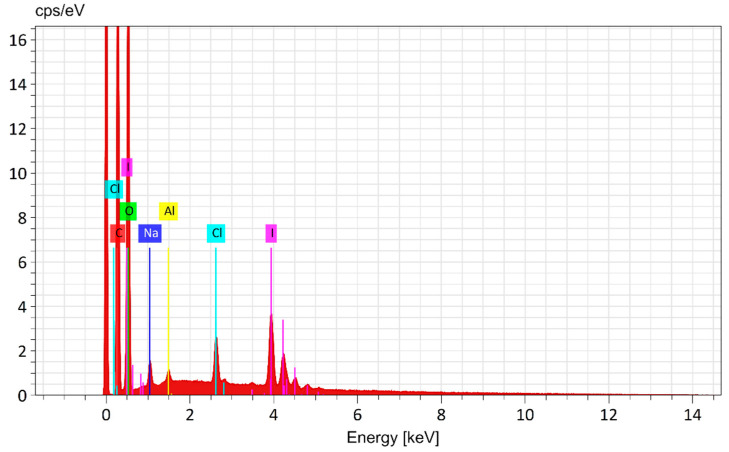
EDX spectrum of the IDLC.

Quantitative elemental analysis of IDLC was performed using a JEOL JSM-IT200 (JEOL, Tokyo, Japan) scanning electron microscope equipped with an energy-efficient X-ray detector (EDX) (Tokyo, Japan). The distribution of the elements was investigated in several directions for greater reliability. The data obtained using EDX analysis consists of spectra showing peaks corresponding to the elements that make up the true composition of the analyzed samples. Elemental mapping of the sample and image analysis are also possible (Figure 5), as shown in Figure 6.

The results of EDX IDLC showed that the mass percentages of carbon, oxygen, chlorine, and iodine were 34.62 ± 0.15; 47.68 ± 0.33; 1.55 ± 0.05; and 10.15 ± 0.16%, respectively. (Table 3). The mass percentages of the constituent elements were compared at all the analyzed points, and it was found that the results were consistent with each other, indicating an equal distribution of the constituent elements in the compound sample. The analysis results confirm the proposed molecular formula of the C_294_H_490_O_245_ · LiI_3_ · LiClI_2_ complex.

### 2.8. Antimicrobial Activity Screening

In the course of this study, a screening of IDLC was carried out against several strains of microorganisms, including *Staphylococcus aureus*, *Escherichia coli*, *Acinetobacter baumannii*, and *Pseudomonas aeruginosa*. The main objective of this work was to determine the minimum bactericidal concentration (MBC) for each of the tested strains and to evaluate their antimicrobial activity. The studies were conducted in accordance with CLSI standards and included both Gram-positive and Gram-negative microorganisms responsible for acute and chronic infectious diseases, characterized by varying levels of resistance to antibiotic drugs.

Antimicrobial activity was evaluated using six reference strains obtained from the American Type Culture Collection (ATCC) and one clinical isolate: *Staphylococcus aureus* ATCC 6538-P, *Staphylococcus aureus* ATCC BAA-39, *Escherichia coli* ATCC 8739, *Escherichia coli* ATCC BAA-196, *Acinetobacter baumannii* ATCC BAA-1790, *Pseudomonas aeruginosa* ATCC 9027, and *Pseudomonas aeruginosa* TA2. The results of the in vitro screening are presented in Table 4.

IDLC demonstrated the following antimicrobial activity results when expressed per substance/per iodide ions: MBC 1.95/0.84 μg/mL against *S. aureus* ATCC BAA-39; 3.91/1.70 μg/mL against *P. aeruginosa* TA2 and *A. baumannii* ATCC BAA-1790; 7.81/3.38 μg/mL against *E. coli* ATCC 196 and *P. aeruginosa* ATCC 9027; 15.63/6.76 μg/mL against *S. aureus* ATCC 6538-P and *E. coli* ATCC 8739. The compound IDLC also exhibits significant activity, especially against the multi-resistant strain *S. aureus* ATCC BAA-39, with an MBC recorded at a concentration of 1.95/0.84 μg/mL; for *A. baumannii* ATCC BAA-1790 and *P. aeruginosa* TA2, the MBC is 3.91/1.70 μg/mL; it inhibits the growth of Gram-negative bacteria at a concentration of 7.81/3.38 μg/mL and has an MBC of 15.63/6.76 μg/mL against *S. aureus* ATCC 6538-P and *E. coli* ATCC 8739, which makes it promising for further research.

### 2.9. Cytotoxicity Study

The study of cytotoxicity is an integral part of the process of developing new anti-infective drugs, as it allows us to assess the safety of potential drugs for living cells.

At the stage of development of new active pharmaceutical substances, it is extremely important to determine their effect on cells in order to avoid potential side effects. Cytotoxicity assessment makes it possible to establish safe doses and regimens of drugs, as well as to prevent possible risks in their clinical use.

Cytotoxicity studies of the IDLC have been conducted on different types of cells, such as human peripheral blood mononuclear cells (PBMCs) and dog kidney epithelial cells (MDCK). Detection of cytotoxicity for various living cells contributes to a more comprehensive assessment of the drug’s safety. Each cell type represents a specific model reflecting different aspects of interaction with the drug, which helps to obtain a more accurate and complete picture of its toxicity. As part of this task, studies have been conducted to study the cytotoxic effect of IDLC on PBMC. A quantitative assessment of the cytotoxic effect of the studied substances was carried out using an MTT test. Cytotoxicity assessment and determination of cytotoxic concentration (CC_50_) of IDLC substances after 24 and 48 h of exposure to PBMC culture in vitro were performed using GraphPad Prism 6 (Table 5).

**Figure 7:**
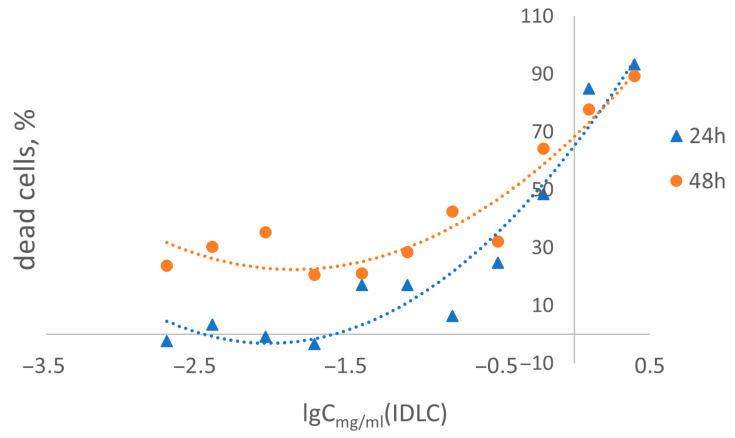
Dose-effect curve for IDLC on PBMC culture after 24- and 48 h exposure.

As a result of the study, curves of the effect dependence on the studied concentrations of IDLC substances on PBMC culture were obtained using the nonlinear regression method (Figure 7). After processing the entire concentration range, the CC_50_ value was calculated in the program, which for IDLC was 0.23 mg/mL at 24 h exposure. With 48 h exposure to CC_50_ IDLC = 0.48 mg/mL. According to the recommendations [20,21], the maximum tolerable (maximum non-toxic) concentration is ¼ CC_50_, respectively, the maximum non-toxic concentration at 24 h exposure to PBMC for substance IDLC is 0.06 mg/mL, at 48 h exposure for IDLC—0.12 mg/mL. Effective doses, as a rule, should be less than these concentrations.

A conversion of the CC_50_ values to the equivalent mass content of elemental iodine (µg I/mL) was performed, taking into account the iodine mass fraction in IDLC (50.71 g/kg). After this conversion, the CC_50_ values for PBMCs were 11.66 µg I/mL (24 h) and 24.34 µg I/mL (48 h).

Studies of the cytotoxicity of IDLC substances in human peripheral blood PBMC culture have shown that the complex exhibits varying degrees of toxicity depending on the concentration and time of exposure.

As part of this task, a study of the safety of substances was also conducted on MDCK cell culture, since studying the safety of IDLC on different cell types can help to obtain a more complete picture of toxicity.

**Figure 8:**
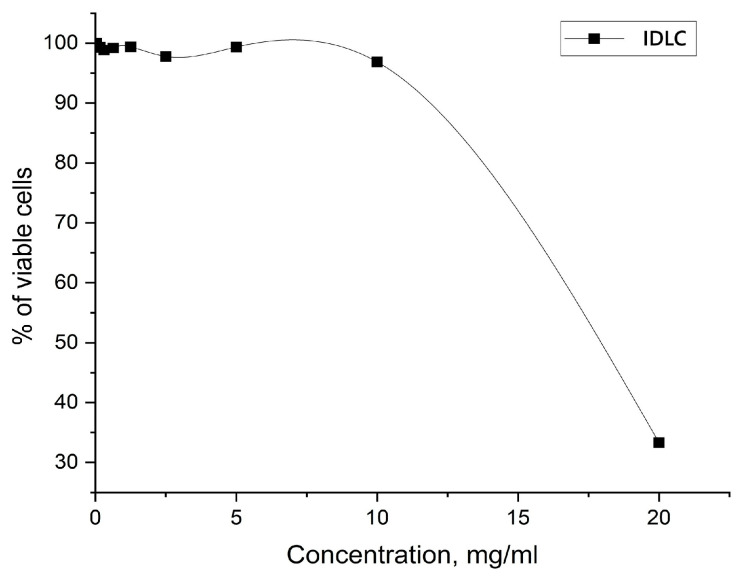
Cytotoxic effect of IDLC on MDCK cell culture.

The cytotoxicity of the substances in vitro was determined using the MTT test. The MDCK cell culture was seeded into 96-well plates at a concentration of 2 × 10^5^ cells per 1 mL. The plates were cultured in a thermostat at 37 °C and 5% CO_2_. The results are presented in Figure 8.

Based on the results obtained to determine the safety of the new complex in in vitro experiments on MDCK cell culture, it was found that IDLC is a low-toxic compound with CC_50_ concentrations ranging from 10.0 to 20.0 mg/mL. Considering that the complex contains 5.071% (*w*/*w*) elemental iodine, the CC_50_ expressed as iodine-equivalent was 507.1–1014.2 µg I/mL.

The data obtained are important for the further development and optimization of the dosage of the studied complex as an API, ensuring a balance between the effectiveness and safety of the drug.

### 2.10. Safety Assessment in Tumor and Normal Cell Lines

To evaluate the in vitro safety of the new IDLC complex, its cytotoxic effects were studied on HeLa, HepG2, AGS, K-562, H9, and normal MeT-5A cell lines.

When exposed to IDLC at a concentration of 5 mg/mL, toxicity was observed against HepG2 and K562 tumor lines (28.7% and 11.8% of viable cells, respectively) and especially AGS (3.9% of viable cells), as well as the normal MeT-5A line (34.9% of viable cells). In relation to the H9 tumor line, the toxic effect was noted at concentrations of 5 and 2.5 mg/mL (9.8% and 38.1% of viable cells, respectively) (Table 6).

After processing the entire concentration range in the program, the CC_50_ value for IDLC was calculated for the HeLa cell line = 1.201 mg/mL (C.I. 0.049 to 29.44), AGS = 1.765 mg/mL (C.I. from 1.600 to 1.947), K562 = 3.533 mg/mL, H9 = 2.003 mg/mL (C.I. from 1.709 to 2.349), and normal MeT-5A cells = 1.370 mg/mL (C.I. from 1.133 to 1.656). After converting the cytotoxicity values to iodine-equivalent (based on iodine content 50.71 g/kg), CC_50_ values ranged from 60.8 to 179.1 µg I/mL across the tested cell lines. The lowest sensitivity was observed in HeLa cells (60.8 µg I/mL), while K562 cells demonstrated the highest tolerance (179.1 µg I/mL).

Thus, the results of the cytotoxicity assessment of IDLC in tumor and normal cell lines indicate the presence of non-specific cytotoxicity at high concentrations, consistent with its iodine content.

## 3. Materials and Methods

### 3.1. Reagents

The reagents used for the present work were of analytical grade, obtained from commercial sources, and used without further purification. Potato starch (JSC “Rogoznitsky Starch Plant”, Lyada, Belarus, 98%), dextrin (Sigma-Aldrich, Steinheim, Germany), lithium iodide (Sigma-Aldrich, Steinheim, Germany, ≥98%), iodine (G. Amphray Lab., Mumbai, India), hydrochloric acid, lithium chloride (Sigma-Aldrich, Steinheim, Germany, ≥99%), purified water by water purification system UltraClear TWF (SG Wasseraufbereitungund Regenerierstation GmbH, Barsbüttel, Germany), sodium thiosulfate, silver nitrate, nitric acid (fixanal, Uralkhiminvest, Moscow, Russia), standard solutions for pH-meter (Reagecon Diagnostics Ltd., Shannon, Ireland).

### 3.2. Test-Systems

The following museum test strains were used in this study: from the WHO list of strains, *Staphylococcus aureus* ATCC 6538-P is a sensitive reference strain for determining antimicrobial activity, obtained from the Republican Collection of Microorganisms (RCM), Astana, Kazakhstan; *Staphylococcus aureus* ATCC BAA-39 is a resistant test strain obtained from the American Collection of Type Cultures (ATCC), Manassas, VA, USA; *Escherichia coli* ATCC 8739 is a reference straina strain for determining antimicrobial activity obtained from the American Collection of Type Cultures (ATCC), USA; *Escherichia coli* ATCC BAA-196 is a resistant test strain for determining antimicrobial activity, obtained from the American Collection of Type Cultures (ATCC), USA; *Pseudomonas aeruginosa* ATCC 9027 is a reference strain for determining antimicrobial activity, obtained from the American Collection of Type Cultures (ATCC), USA; *Pseudomonas aeruginosa* TA2 is a resistant strain from clinical study; and *Acinetobacter baumanii* ATCC BAA-1790 is a resistant test strain for determining antimicrobial activity obtained from the American Collection of Type Cultures (ATCC), USA.

To conduct safety and efficacy studies of the new active compound in vitro, the following research objects were used:

Human blood—the work used whole peripheral blood from healthy (absence of acute diseases and severe chronic diseases) donors of both sexes as a source of human immunocompetent cells.

Cell lines—H9 (human T cell lymphoma, ATCC-HTB-176, USA), K-562 (human erythroblastoid leukemia, ATCC-CCL-243, USA), HepG2 (human hepatocellular carcinoma, ATCC-HB-8065TM, USA), AGS (human gastric adenocarcinoma cells, ATCC-CRL-1739, USA), HeLa (human cervical adenocarcinoma, ATCC-CCL-2, USA), MeT-5A (SV40—immortalized human mesothelial cell line, ATCC-CRL-9444, USA) were used for studying the antitumor effect.

Cell cultures—MDCK kidney cells of the Madin–Darby Canine Kidney dog, obtained from the Laboratory of Cellular Biotechnology of the Research Institute of Problems of Biological Safety of the National Research Library of the Ministry of Education and Science of the Republic of Kazakhstan.

### 3.3. Preparation of the Iodine-Containing Complex

#### 3.3.1. Preparation of Dextrin Solution

A starch solution was prepared to obtain dextrin. A flat-bottomed glass flask was filled with demineralized, distilled water to a certain amount. Next, starch powder measured on an electronic scale was added to the flask. The solution was thoroughly mixed for 30 min. Then, it was poured into a working solution of hydrochloric acid, which was 1/3 of the calculated volume. Then, the starch solution (with a molecular weight of 7846 kDa) was transferred to a glass reactor. Hydrolysis was carried out for 25 min at a temperature not lower than 88 °C with constant stirring. At this stage, the temperature control of the process was carried out using a combined Testo 925 measuring device. When the required state was reached, a visual change in the solution to a more transparent one was observed.

#### 3.3.2. Preparation of a Solution of Lithium Triiodide

A glass flask with a volume of 500 mL was filled with distilled water; then, a measured amount of lithium iodide was added. After its dissolution, iodine was added after 10–15 min [22]. The contents of the flask are thoroughly mixed. In another flask, the same actions were performed for the magnesium triiodide solution.

#### 3.3.3. Preparation of Lithium Chloride Solution

The required amount of lithium chloride was weighed in an analytical balance with an accuracy of ±0.001 g. It was then dissolved with distilled water and mixed until completely dissolved.

#### 3.3.4. Mixing Solutions

The prepared solutions of the components were combined into one flask with constant stirring to avoid local concentration differences. The mixture was poured into a mold, which was pre-washed and dried.

#### 3.3.5. Crystallization

The crystallizer with the solution was placed in a desiccator with calcium chloride to ensure slow evaporation of water. The system was left alone until the crystals were completely formed.

### 3.4. Quantum-Chemical Calculations

Geometry optimization and formation energy calculations of the studied structures were performed using the DFT method with the B3PW91 functional and 6-31G** basis set (Wallingford, CT, USA). All computations were carried out on a Fujitsu PRIMERGY BX920 S1 supercomputer (Fujitsu, Tokyo, Japan) with a peak performance of 10.9 TFLOPS. The formation energy of the complex was calculated according to the following expression:

∆E = E_1_ ^total^ − (E_2_ ^total^ + E_3_ ^total^ + E_4_ ^total^), (1)

where E_1_ ^total^—total energy of complex;

E_2_ ^total^—total energy of dextrin helix with polypeptide;

E_3_ ^total^—total energy of Li^+^ ion;

E_4_ ^total^—total energy of I_3_^−^ or I_2_Cl^−^ ion.

### 3.5. UV-Vis Spectroscopy

UV-vis spectra of the samples were collected using a LAMBDA-35 UV-Vis spectrophotometer (PerkinElmer, Waltham, MA, USA). The samples were dissolved in a water solution (1 mg/mL), and the solvent was used as a reference. The scanning range was 190–1100 nm [23].

### 3.6. ^1^H NMR

^1^H NMR spectra of the samples were collected using a superconducting Fourier NMR spectrometer JNM-ECA 500 (JEOL, Tokyo, Japan) operating at 500 MHz, and the solvent used was DCl/D_2_O (1/100, *V*/*V*) [17,18,19].

### 3.7. XRD

The samples were analyzed using an XRD diffractometer, SmartLab (Rigaku Ultima IV, Tokyo, Japan), under the following conditions: Cu Kα radiation (λ = 1.54059 Å), a one-dimensional detector (D/teX Ultra, Rigaku, Tokyo, Japan) with a Kβ filter, and step-scan measurements conducted within a 2θ range of 5–90°, with a step width (Δ2θ) of 0.1° and a scanning speed of 5°/min. Phase identification and investigation of the crystalline structure were performed using the PDXL Version 2.8.4 Integrated X-Ray Powder Diffraction Software and the international database ICDD PDF-2.

### 3.8. Thermogravimetric (TG) Analysis

Thermal stability analysis of the samples was performed using a TG/DSC STA 449 F1 Jupiter (NETZSCH, Selb, Germany) under the following conditions: nitrogen as the carrier gas, a flow rate of 50 mL/min, a temperature range of 30–600 °C, and a heating rate of 10 °C/min [24].

### 3.9. Capillary Electrophoresis (CE)

Capillary electrophoresis was carried out using an Agilent 1600 system with a diode-array detector. Analyte concentrations in sample solutions were adjusted to 5–200 mg/L and filtered through 0.2 µm membranes. Qualitative identification was based on migration time comparison with reference standards, while quantitative determination relied on peak area analysis against certified standards [20].

### 3.10. SEM-Analysis

SEM analysis was conducted using a Hitachi TM4000Plus tabletop scanning electron microscope (Hitachi, Tokyo, Japan) equipped with an energy-dispersive X-ray spectroscopy (EDX) module. Imaging was performed at a magnification of ×500 under an accelerating voltage of 15 kV.

### 3.11. Method of Antimicrobial Activity Screening

To determine the MICs of each antimicrobial agent individually, a broth microdilution assay was performed using the method of two-fold serial dilutions according to the CLSI (Clinical and Laboratory Standards Institute) protocol. The bacterial suspension was adjusted to a turbidity of 0.5 McFarland units, corresponding to approximately 1.5 × 10^8^ CFU/mL. For preparation of the working bacterial suspensions, the stock inoculum was diluted 100-fold in isotonic saline to obtain a final concentration of approximately 1.5 × 10^6^ CFU/mL.

Microtiter plates were prepared stepwise as follows:

1. 100 µL of appropriate liquid growth medium was added to the required wells.
2. 100 µL of a pre-prepared 4× stock solution of the corresponding antibiotic was added to the first wells of each row (A1, B1, C1, D1, E1, F1, and H1), followed by two-fold serial dilutions across the row to create horizontal serial dilutions of the antibiotic.
3. 100 µL of pre-prepared 2× dilutions of the APS compound were added vertically to generate serial dilutions along the columns.
4. 20 µL of the working inoculum solution was added to each well containing 200 µL of the antibiotic and compound mixture. As a result, the final bacterial concentration in each well after inoculation was approximately 1.5 × 105 CFU/mL.

Control wells included a growth control (medium with inoculum, without test compounds) and a sterility control (medium only, without inoculum).

The experiments were performed in three fully independent biological replicates, and each of these included technical replicates.

Plates were incubated for 18–24 h at (37 ± 1) °C (Binder, Tuttlingen, Germany). After the incubation period, 30 µL of 0.01% aqueous resazurin solution was added to all wells of the experimental plates [21,25]. The results were assessed by monitoring the color change of the resazurin indicator, which signified the absence of metabolic activity and, consequently, the absence of bacterial growth. In all cases, wells that showed no signs of metabolic activity were additionally plated onto solid nutrient media and incubated to confirm the absence of bacterial growth. Thus, the MBC values were determined using both the resazurin read-out and confirmatory plating onto solid Mueller–Hinton agar.

### 3.12. Cytotoxic Effect

#### 3.12.1. Mononuclear Cells

Peripheral whole blood from healthy male and female donors (free from acute and severe chronic diseases) was used as a source of human immunocompetent cells. Peripheral blood mononuclear cells (PBMCs) were isolated by density gradient centrifugation using Histopaque (Sigma, St. Louis, MO, USA) at a 1:1 ratio, followed by centrifugation at 3000 rpm for 20 min at 4 °C. The mononuclear cell fraction was washed twice and resuspended in RPMI-1640 culture medium (Sigma, St. Louis, MO, USA). Cell viability was determined by trypan blue exclusion, and only samples with >90% viable cells were used for further experiments [21,25]. The CC_50_ values were calculated using GraphPad Prism 6 with a nonlinear regression model that incorporates the entire concentration range. The total iodine content in IDLC is 50.71 g/kg (5.071% *w*/*w*). To standardize the toxicological parameters, the CC_50_ values were converted to iodine-equivalent (µg I/mL) using the following formula:

CC_50_I(μg/mL) = CC_50_complex(mg/mL) × w_I_ × 1000, (2)

where w_I_ = 0.05071.

#### 3.12.2. Madin–Darby Canine Kidney Cells

MDCK (Madin–Darby Canine Kidney) cells, derived from canine kidney epithelium, were obtained from the Cell Biotechnology Laboratory of the Research Institute for Biological Safety Problems, National Center for Biotechnology. The cells were seeded into 96-well plates at a density of 2 × 10^5^ cells/mL and incubated at 37 °C in a humidified atmosphere containing 5% CO_2_.

In vitro cytotoxicity of the compound was evaluated using the MTT assay. Compound was added once to the wells containing the cell suspension at final concentrations of 20.0, 10.0, 5.0, 2.5, 1.25, 0.63, 0.31, 0.16, 0.08, and 0.04 mg/mL. The exposure duration was 72 h. Cytotoxic effects were assessed based on cell viability in comparison to untreated controls [21,25].

#### 3.12.3. MTT Assay

The MTT assay is based on the ability of living cells to reduce the soluble yellow tetrazolium salt 3-(4,5-dimethylthiazol-2-yl)-2,5-diphenyl tetrazolium bromide (MTT) into insoluble purple-blue intracellular formazan crystals. Non-viable cells lack this capacity. Four hours before the end of exposure to the tested compounds, 10 μL of MTT solution was added to each well, followed by incubation for an additional 4 h in a CO_2_ incubator at 37 °C, 5% CO_2_, and 95% humidity.

The resulting formazan crystals were dissolved in 100 μL DMSO, and the optical density (OD) was measured using a Sunrise RC microplate reader at wavelengths of 492 or 540 nm.

It should be noted that the MTT assay measures metabolic activity, and iodine-containing compounds may directly influence MTT reduction. Therefore, the observed OD values reflect cell viability with possible interference from iodine rather than exclusively cell death [26].

## 4. Conclusions

Comprehensive physicochemical, spectroscopic, and biological analyses confirm the successful synthesis and stability of a novel hybrid supramolecular system: the iodine–dextrin–lithium complex (IDLC). Theoretical modeling using DFT calculations demonstrated a highly favorable formation energy (ΔE = −843.27 kcal/mol), indicating strong thermodynamic stability and efficient coordination between Li^+^, polyiodide species, and dextrin hydroxyl groups. The optimized geometry reveals chelating interactions (Li-O = 2.22 Å, Li-I = 3.02 Å), which ensure the spatial fixation of iodine within the polysaccharide matrix and support controlled release alongside enhanced structural order.

Spectroscopic investigations (UV-Vis, ^1^H/^13^C NMR) and XRD analyses confirmed the formation of a semi-crystalline supramolecular structure, where iodine species exist in both bound and crystalline states. Characteristic absorption bands at 194–450 nm, chemical shift deviations in NMR spectra, and diffraction peaks corresponding to LiI and LiI-I_2_ phases collectively indicate complexation between iodine, lithium, and dextrin via noncovalent and coordination interactions. Thermal analysis revealed a two-stage degradation process, with a melting point of 169.6 °C and a total mass loss of approximately 61.5%, confirming the high purity and improved thermal stability of the complex compared to native dextrin.

When expressed in iodine-equivalent units, the MBC values of IDLC were substantially lower than those reported for classical povidone-iodine (PVP-I). Published MIC/MBC values for PVP-I against bacteria generally fall within 1000–5000 µg/mL of available iodine, depending on formulation and assay conditions. For instance, Barakat, N. A. et al. reported an MBC of approximately 5000 µg/mL [27], while Lux et al. demonstrated bactericidal activity at 1250 µg/mL available iodine [28]. Comparative antiseptic studies, such as that of Koburger et al., have reported MBC values up to 1024 mg/L for several Gram-positive strains [29]. Collectively, these data consistently indicate a significantly lower bactericidal potency of PVP-I compared with the iodine-equivalent values obtained for IDLC.

The specific mechanisms underlying the enhanced antimicrobial activity of IDLC remain to be elucidated. While lithium ions have been reported to influence membrane fluidity, osmotic balance, and cellular stress responses in microorganisms [30], the present study does not directly demonstrate a mechanistic contribution of lithium to iodine’s bactericidal effect. Therefore, any proposed synergistic interactions should be considered preliminary. Lithium may function primarily to stabilize the complex or to modulate the release of active iodine species rather than exerting an independent antimicrobial role.

Overall, the iodine-equivalent MBC values obtained for IDLC are markedly lower than the reported values for PVP-I and ILαD. These findings indicate a potentially higher antimicrobial potency of the new complex. However, further investigations-including direct comparisons with lithium-free dextrin–iodine complexes, analysis of iodine release kinetics, and mechanistic studies-are essential to clarify the nature of this enhanced activity and to determine whether lithium contributes directly to improved bactericidal performance.

Cytotoxicity assays revealed moderate, concentration-dependent effects on human mononuclear cells (CC_50_ = 0.23–0.48 mg/mL IDLC, corresponding to 11.7–24.3 μg I/mL) and low toxicity toward MDCK cells (CC_50_ = 10–20 mg/mL IDLC, corresponding to 507–1014 μg I/mL), indicating a favorable therapeutic index. The calculated maximum non-toxic concentrations (0.06–0.12 mg/mL IDLC, 3–6 μg I/mL for PBMCs) further delineate a broad safety margin for future pharmacological evaluation.

IDLC also exerts cytotoxic effects on tumor cell lines (HepG2, HeLa, AGS, K562, and H9) and normal MeT-5A cells; however, the CC_50_ values are similar, and the selectivity indices are close to 1, indicating no selective cytotoxicity toward tumor cells. Thus, IDLC demonstrates non-specific cytotoxicity at high concentrations, consistent with its iodine content.

Comparison with published data shows that PVP-I at relatively low concentrations (≥0.5%) rapidly kills mesothelial and tumor cells within a short time (5–60 min); for example, the IC_50_ by MTT for the MeT-5A cell line is approximately 0.3–0.32% PVP-I [31,32]. Similarly, in a comparative study of ILαD and PVP-I, it was found that at concentrations corresponding to the minimum bactericidal dose (MIC), ILαD causes only a moderate reduction in cell viability, whereas PVP-I results in 90–95% cell death [12]. A limitation of the present study is that cytotoxicity was assessed solely using the MTT assay. Since iodine can directly interfere with MTT reduction, some observed effects may represent assay artifacts rather than true cell death. Future studies will confirm these findings using complementary methods, such as LDH release, Annexin V/PI staining, or SRB assay, to more accurately assess cytotoxicity.

The integration of iodine, lithium, and dextrin into a single supramolecular system has yielded a chemically stable, thermally resistant, and biologically active complex. From a pharmaceutical perspective, the IDLC demonstrates promising characteristics as a biocompatible antiseptic and stabilizing platform suitable for development into next-generation iodine-based pharmaceutical formulations with enhanced efficacy against multidrug-resistant pathogens.

## Figures and Tables

**Table 1:** Physicochemical properties of the dextrin/iodine complexes.

Compound	Solubility in Water	pH	Melting Point, °C	Quantitative Determination of Iodine, g/kg
IDLC	1 g/20 mL, soluble	4.83 ± 0.01	146–148 ± 1	50.71 ± 0.01

**Table 2:** Quantitative determination of iodide ions and cations by the CE method.

Compound	I^−^, mg/L (CE)		Li^+^, mg/L (CE)	
	Exp.	Theo.	Exp.	Theo.
IDLC	43.25 ± 0.08	47.5	6.78 ± 0.01	8.2

**Table 3:** EDX analysis data of IDLC.

Element	Mass % (Theory) C_294_H_490_O_245_ · LiI_3_ · LiClI_2_	Mass % (Detected)	Line Type
**C**	40.92	34.62 ± 0.15	K Series
**O**	45.43	47.68 ± 0.33	K Series
**Cl**	0.41	1.55 ± 0.05	K Series
**I**	7.35	10.15 ± 0.16	L Series
Li *	0.16	-	-
H *	5.72	-	-
**Totals**	100	94.3	

* The numbers of H and Li are calculated from the difference in the mass of the sample and the elements determined in the experiment. Na and Al were treated as impurities and excluded from the quantitative comparison between theoretical and experimental elemental mass fractions.

**Table 4:** Results of antimicrobial activity screening of the IDLC.

Compound Name	Name of the Test Strains						
	*S. aureus ATCC 6538-P*	*S. aureus ATCC BAA-39*	*E. coli ATCC 8739*	*E. coli ATCC 196*	*P. aeruginosa ATCC 9027*	*P. aeruginosa TA2*	*A. baumannii ATCC BAA-1790*
	Value of the Minimum Bactericidal Concentration (MBC), μg/mL in Terms of Substance/Iodide Ions						
IDLC	15.63/6.76	1.95/0.84	15.63/6.76	7.81/3.38	7.81/3.38	3.91/1.70	3.91/1.70

**Table 5:** Cytotoxic effect of IDLC on human peripheral blood PBMC culture after 24- and 48 h exposure.

Concentration of IDLC, mg/mL	% of Viable Cells (Mean ± SD)	
	24 h	48 h
Negative control	100.0 ± 13.4	100.0 ± 13.0
2.5	6.7 ± 4.7	10.8 ± 1.8
1.25	15.0 ± 4.5	22.3 ± 29.8
0.625	51.5 ± 15.5	35.8 ± 5.5
0.312	75.2 ± 22.2	57.9 ± 14.2
0.156	83.6 ± 9.7	59.6 ± 4.8
0.078	83.0 ± 12.5	71.6 ± 13.1
0.039	83.0 ± 22.4	78.9 ± 17.1
0.019	103.3 ± 22.6	79.4 ± 15.9
0.009	100.8 ± 23.0	64.8 ± 6.6
0.004	96.6 ± 15.9	69.8 ± 7.5
0.002	122.3 ± 29.0	76.2 ± 15.5
CC_50_ IDLC (mg/mL)	0.23	0.48
CC_50_I (μg/mL)	11. 66	24.34

**Table 6:** Cytotoxicity of IDLC on tumor and normal cell lines in vitro.

Concentration, mg/mL	% of Viable Cells (Mean ± SD)					
	HepG2	HeLa	AGS	K562	H9	MeT-5A
Negative control	100.0 ± 6.5	100.0 ± 3.7	100.0 ± 3.8	100.0 ± 3.0	100.0 ± 2.3	100.0 ± 4.6
5	28.7 ± 2.5	50.6 ± 2.7	3.9 ± 0.6	11.8 ± 2.4	9.8 ± 0.5	34.9 ± 2.8
2.5	74.9 ± 9.7	102.4 ± 3.3	28.0 ± 0.4	108.2 ± 4.6	38.1 ± 4.1	50.0 ± 0.8
1.25	158.6 ± 8.6	182.9 ± 5.3	88.9 ± 0.6	98.6 ± 13.9	92.3 ± 9.8	81.7 ± 3.0
0.625	154.4 ± 2.7	142.1 ± 4.3	101.6 ± 3.3	97.5 ± 4.1	95.4 ± 4.7	123.1 ± 3.0
0.312	130.2 ± 11.4	136.0 ± 2.9	101.6 ± 3.3	103.3 ± 3.6	95.1 ± 8.1	117.8 ± 6.9
0.156	129.0 ± 6.4	129.9 ± 1.4	111.7 ± 4.0	101.2 ± 5.9	103.8 ± 10.5	110.9 ± 1.9
0.08	124.0 ± 4.7	116.1 ± 1.7	102.4 ± 0.7	95.8 ± 11.9	87.4 ± 6.9	114.5 ± 5.4
0.04	125.9 ± 8.4	136.5 ± 1.7	106.3 ± 3.6	89.9 ± 8.2	98.0 ± 17.7	113.1 ± 4.3
0.02	149.0 ± 31.2	113.5 ± 9.3	98.8 ± 7.0	85.6 ± 3.7	95,7 ± 4.0	99.3 ± 16.9
CC_50_ IDLC (mg/mL)	~2.440	1.201	1.765	3.533	2.003	1.370
CC_50_I (μg/mL)	123.7	60.8	89.5	179.1	101.6	69.5

## Data Availability

The data used to support the findings of this study are available from the corresponding authors upon reasonable request.

## Supplementary Materials

The following supporting information can be downloaded at: https://www.mdpi.com/article/10.3390/molecules30244822/s1, Figure S1: ^1^H NMR spectra of the IDLC sample; Figure S2: ^13^C NMR spectra of the IDLC sample; Figure S3: Possible structural formula of IDLC.

## Abbreviations

The following abbreviations are used in this manuscript:

API	Active pharmaceutical ingredient
CC_50_	Half-cytotoxic concentration
DSC	Differential scanning calorimetry
IDLC	Iodine–dextrin–lithium complex
MBC	Minimum bactericidal concentrations
MDCK	Madin–Darby Canine Kidney
MTT	3-(4,5-dimethylthiazol-2-yl)-2,5-diphenyltetrazolium bromide
CE	Capillary electrophoresis
PBMCs	Fixed Mononuclear cells on Peripheral blood mononuclear cells
XRD	X-ray diffraction analysis
