# Supplementary figures and images for: The Iodine–Dextrin–Lithium Complex: Morphology, Antibacterial Activity, and Cytotoxicity
Source: Molecules. 2025 Dec 18;30(24):4822. doi: 10.3390/molecules30244822 (PMC12736063; doi:10.3390/molecules30244822)

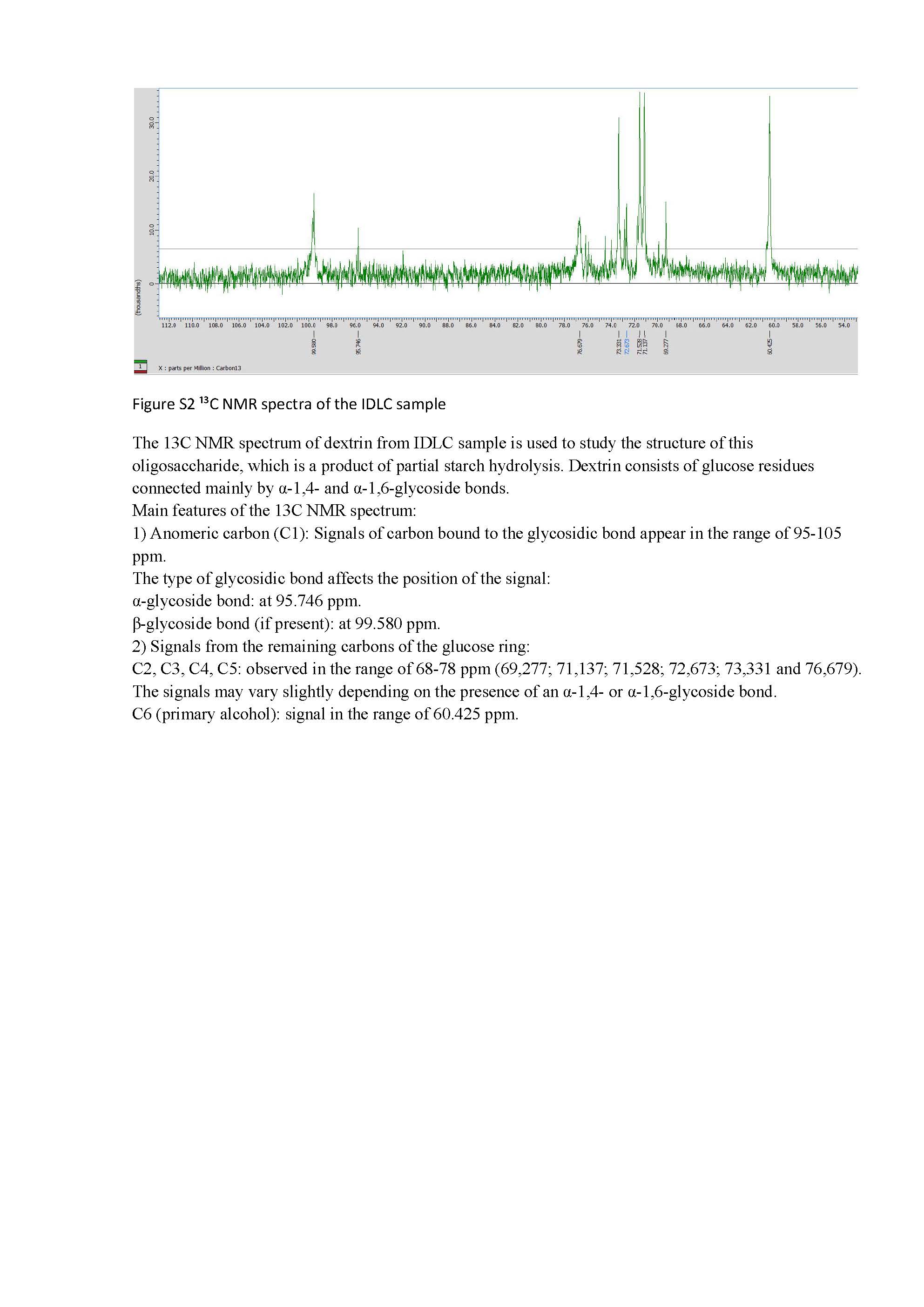

Supplement: Supplementary file 1 [file molecules-30-04822-s001.zip › Fig S2 13C NMR.jpg]

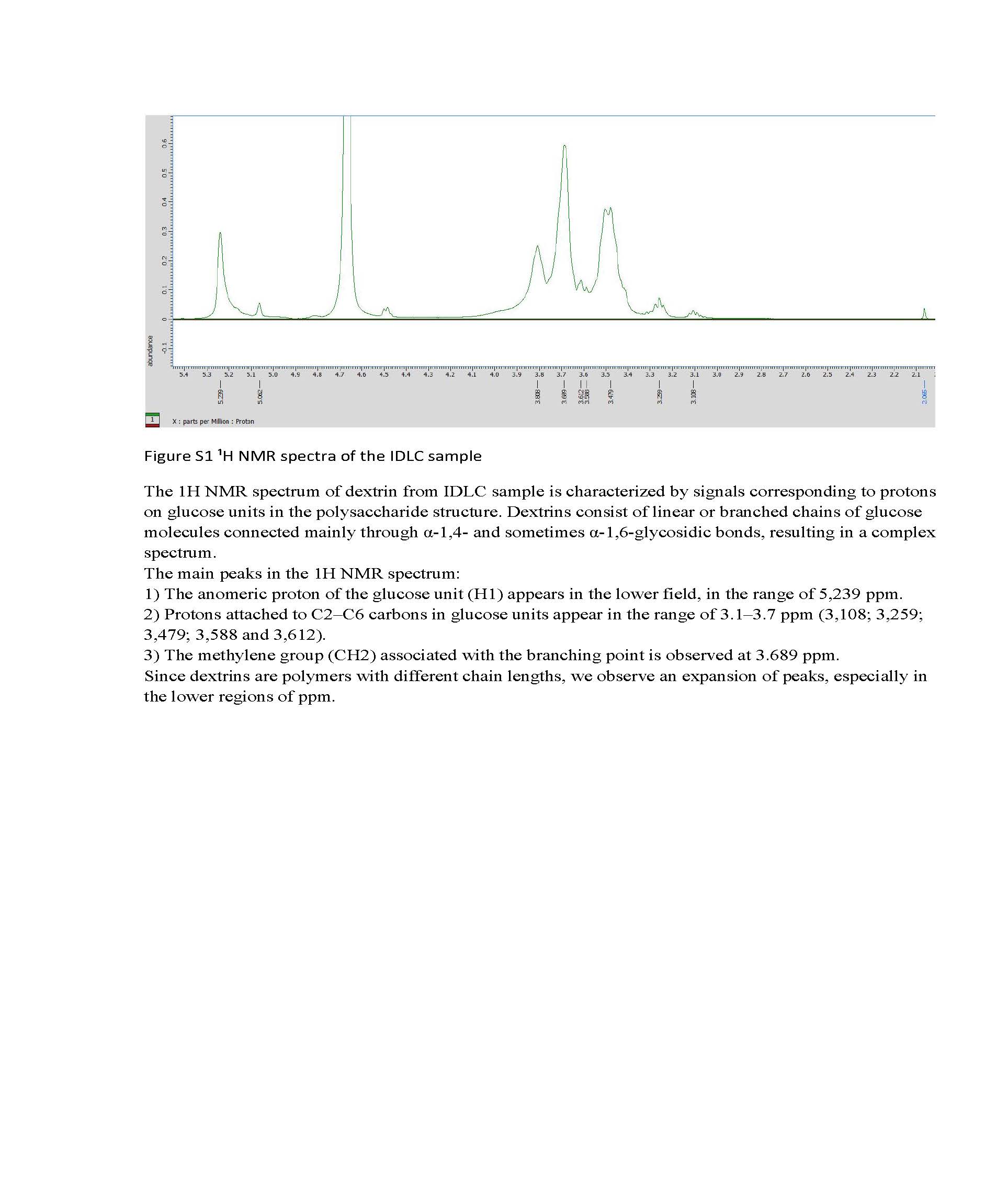

Supplement: Supplementary file 1 [file molecules-30-04822-s001.zip › Fig S1 1H NMR.jpg]

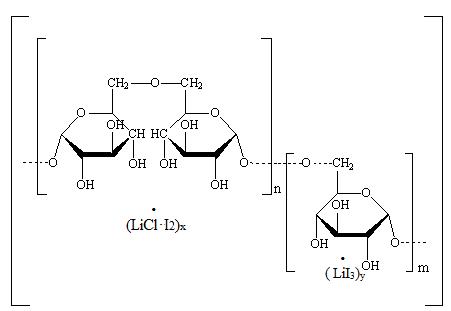

Supplement: Supplementary file 1 [file molecules-30-04822-s001.zip › Fig S3 Possible structural formula of IDLC.jpg]
